# The need for co-educators to drive a new model of inclusive, person-centred and respectful co-healthcare with people with intellectual disability

**DOI:** 10.3389/fpsyt.2024.1346423

**Published:** 2024-02-13

**Authors:** Chloe Molnar, Iva Strnadová, Manjekah Dunn, Julie Loblinzk, Skie Sarfaraz, Yasmin Cathcart-King, Michelle Tso, Joanne Danker, Sarah Hayes, Sierra Angelina Willow, Jennifer Hansen, Tiffany Qing Lim, Jackie Boyle, Bronwyn Terrill, Jackie Leach Scully, Elizabeth Emma Palmer

**Affiliations:** ^1^ Discipline of Paediatrics and Child Health, Faculty of Medicine and Health, University of New South Wales, Sydney, NSW, Australia; ^2^ School of Education, University of New South Wales, Sydney, NSW, Australia; ^3^ Disability Innovation Institute, University of New South Wales, Sydney, NSW, Australia; ^4^ Self-Advocacy Sydney Inc., Sydney, NSW, Australia; ^5^ Centre for Clinical Genetics, Sydney Children’s Hospitals Network, Sydney, NSW, Australia; ^6^ The New South Wales Genetics of Learning Disability (GOLD) Service, Waratah, NSW, Australia; ^7^ Australian Genomics, Melbourne, VIC, Australia; ^8^ Kinghorn Centre for Clinical Genomics, Garvan Institute of Medical Research, Darlinghurst, NSW, Australia; ^9^ School of Clinical Medicine, Faculty of Medicine and Health, University of New South Wales, Sydney, NSW, Australia

**Keywords:** intellectual disability, co-production, inclusion, healthcare for consistency, education

## Introduction

According to the medical model, intellectual disability is a neurodevelopmental condition of childhood onset that presents with limitations in intellectual and adaptive functioning (1). However, as emphasised by Robert Strike, recipient of the Medal of the Order of Australia (OAM) and co-founder of Self Advocacy Sydney, an organisation that empowers people with intellectual disability to speak up for themselves, “Intellectual disability is not an inability to think!” (2, 3). Intellectual disability is also not an inability to feel and remember experiences, as evidenced by GeneEQUAL research (4). GeneEQUAL is an inclusive research program at the University of New South Wales, Sydney, that aims to improve genetic healthcare for people with intellectual disability (5).

## Healthcare rights

There is growing emphasis on person-centred care that recognises an individual’s capability and potential to manage their own health and involves shared decision-making between patients and health professionals (6, 7). This can improve physical and psychological health outcomes and increase individuals’ skills and confidence in managing their own health (6). The United Nations (UN) *Convention on the Rights of Persons with Disabilities* (CRPD) states that people with disability should have “full and equal enjoyment of all human rights” without discrimination, including the right to the highest attainable standard of health, which includes person-centred care (Article 25) and equal access to medical facilities and information (Article 9) (8). Currently, 164 countries, including Australia, have signed the Convention (9), and some countries have additional legislation aiming to reduce stigma and discrimination in the healthcare setting (10–12). Examples include the *Equality Act 2010* in the United Kingdom and the *Disability Discrimination Act 1992* in Australia, both of which require healthcare professionals to make reasonable adjustments to improve accessibility (10, 12). Reasonable adjustments are changes that do not impose an undue burden but ensure that people are not disadvantaged or harmed (11, 13). They can include adapting communication to meet a person’s needs, providing information in alternative formats including Easy Read, and allowing extra time to share information and provide support (13).

## Stigma and abuse in healthcare

People with intellectual disability continue to experience high levels of stigma, resulting in a denial of equal rights, psychological distress (14, 15), and a disproportionately high risk of physical, sexual, emotional, financial, and disability-related abuse (16, 17). This extends to healthcare settings, and GeneEQUAL co-researchers shared their adverse and often traumatic experiences of neglect and abuse, including not having the opportunity to make their own healthcare decisions, being ignored when they present with a support person, and feeling pressured to provide consent (18). In Australia, the 2023 *Royal Commission into Violence, Abuse, Neglect and Exploitation of People with Disability* (the Disability Royal Commission) found ongoing systemic neglect and abuse of people with intellectual disability within the health system (19). An additional study surveying over 600 intellectual disability experts and organisations across 88 countries showed that people with intellectual disability in low- and middle-income countries were often denied human rights and freedoms and experienced high levels of sigma (14), including in healthcare (20). People with intellectual disability mostly relied on family for support, and, in some countries, it was usual to segregate people with intellectual disability from society (14).

Stigma and trauma in healthcare are also directly associated with poor health outcomes (21, 22). During hospitalisation, people with intellectual disability commonly experience worse care (23) and have longer inpatient stays compared with the general population (24). The Australian Government’s *National Roadmap for Improving the Health of People with Intellectual Disability* highlighted that people with intellectual disability face barriers accessing safe and quality care, evidenced by significantly lower rates of preventative healthcare, including regular health checkups and screening for disease, more than double the rate of avoidable mortality, and twice the rate of emergency department and hospital admissions compared to the general population (25). This mirrors findings in other countries, for example, a Confidential Inquiry, commissioned by the Department of Health in England and conducted between 2010 and 2012 in England and Wales, concluded that there was a higher risk of avoidable deaths amongst people with intellectual disability that could be attributed to untreated illness and poor quality healthcare (26). There is also evidence that people with intellectual disability have less access to information and reduced awareness of their healthcare rights than people without intellectual disability (21). Moreover, such negative healthcare experiences can reduce patient expectations and engagement, further contributing to poor health outcomes and setting up a vicious cycle (27).

### Factors contributing to stigma and abuse at a system level

At a system level, there is limited availability of accessible health information, such as Easy Read and multimodal health resources (including videos, booklets, and websites), and often poor access to alternative communication resources (including sign language interpreters and assistive communication aides) (21). A study looking at 32 consultations between people with intellectual disability and a primary care physician found that only six consultations included the use of Easy Read documents (28). In addition, non-inclusive clinical environments can form major barriers to equitable healthcare, including appointment times that are too short to allow for effective health communication and shared decision-making (29), unwelcoming hospital environments (19, 27), and inadequate integration of healthcare services and continuity of care (30).

### Factors contributing to stigma and abuse at a clinician level

Limited knowledge amongst clinicians about the lived experiences of people with intellectual disability is a major barrier to the delivery of accessible, inclusive, and respectful healthcare (21, 31). A systematic review of 30 studies of various study design revealed that some health professionals considered people with intellectual disability to be unlike other patients and, at times, to be childlike, strange, or intimidating (31). People with intellectual disability also commonly reported experiencing stigma and discrimination from health professionals, including being made to feel inferior, pitied, or over-valorised for their disability (21). In Australia, public hearings held as part of the Disability Royal Commission found that some health professionals made assumptions about the quality of life of people with disability, which could restrict access to high quality healthcare services (19). In addition, because of poor understanding of underlying intellectual disability-related conditions, some health professionals assumed that new symptoms were related to a person’s intellectual disability rather than a new condition (i.e., diagnostic overshadowing), resulting in incorrect or delayed diagnoses and negatively impacting care received (19, 32).

Effective communication is vital for equitable care (27) and health literacy (21) but is often poor in consultations with people with intellectual disability (19, 30). Health professionals are generally unaware of communication challenges faced by people with intellectual disability and how best to modify communication approaches to support inclusion (27). As health professionals often rely on the family and/or support person, health information may not be adequately explained to the individual patient, reducing opportunities for empowerment and shared decision-making (27). This can lead to people with intellectual disability being ignored, contributing to feelings of stress and vulnerability, and this is an overt form of stigmatisation (24, 27). There is also a perception that health professionals may avoid direct communication with people with intellectual disability due to limited understanding of individual needs and the fear that it would entail additional workload, which they are reluctant to take on (24).

## The need for improved clinician education

Minimal clinician knowledge reflects limited teaching about intellectual disability to health students and professionals (24, 32, 33). Australian medical curricula include little education about the healthcare needs of, and minimal exposure to, people with intellectual disability (33). There are also no mandatory training requirements for health professionals to improve their knowledge and skills when working with people with intellectual disability (32), and many health professionals are unaware of relevant legislation (24). Furthermore, a 2017 study of medical schools in the United States estimates that less than a quarter of all medical schools include a disability awareness program (34).

**Figure 1:**
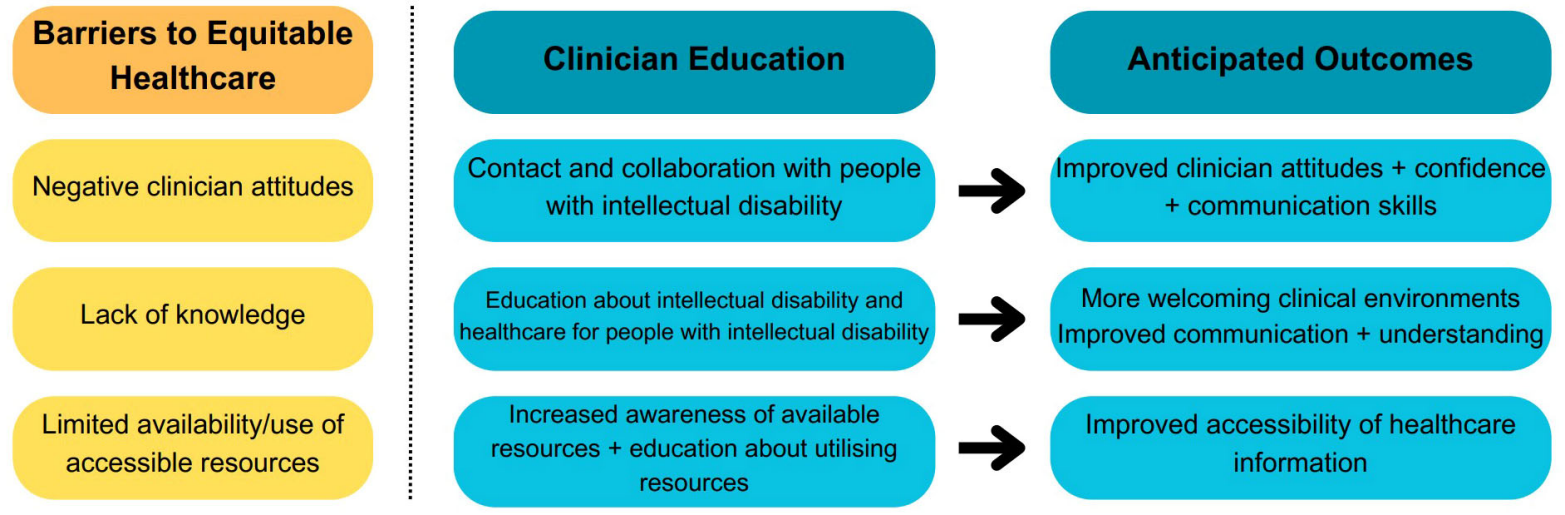
Summary of the impact of education on addressing barriers to equitable healthcare for people with intellectual disability. The arrow represents how aspects of clinician education programs might reduce healthcare barriers (created using https://www.canva.com/).

Well-designed educational programs have the potential to improve clinician capabilities, knowledge and skills in delivering accessible, person-centred, and respectful healthcare for people with intellectual disability (29). Effective clinical educational programs could, therefore, be a powerful way to reduce the stigma that people with intellectual disability currently face in the healthcare system (**Figure 1**) (29). The World Health Organisation Disability-Inclusive Health Services Toolkit recommends that education programs about disability and disability-inclusive healthcare are included in medical school curricula, are a requirement for accreditation, and are offered as training to health professionals (21). In addition, the UN policy guidelines included in a Sustainable Development Goals–CRPD resource package recommend that health professionals receive training to develop skills, improve attitudes, and learn about the rights of people with intellectual disability and how to provide reasonable adjustments (35). Increased knowledge can reduce stigma and allow health professionals to respond to the needs of people with intellectual disability (24), for example, by adapting the hospital environment (23), avoiding diagnostic overshadowing, and communicating effectively (21). Finally, in view of the recognised frequency of trauma and abuse experienced by people with intellectual disability (36), it is also critical that clinician education also incorporates how to deliver trauma-informed care (37).

### Co-production and co-education

The GeneEQUAL team wholeheartedly support the recommendation that educational programs should be co-produced and co-delivered with people with intellectual disability, as they are the experts in their own experiences (29, 38). Co-production involves collaboration between clinical educators and healthcare recipients, in this case, people with intellectual disability, to design and deliver educational programs (39) and to ensure that their opinions and preferences are incorporated (40). Involving people with intellectual disability in the design and delivery of content for medical students and health professionals has been demonstrated to bring transformative change to the individuals involved, as well as to health professionals and health systems (41). A global systematic review looking at patient engagement across all health services only found a small number of studies that involved patients in the co-production of educational programs (42). However, it was evident that co-production led to improved healthcare quality and outcomes and ultimately also improved health governance, policies, and organisational planning. Co-production has also been associated with improved attitudes towards and awareness of the needs of people with intellectual disability (31), improved communication skills (33), and the sharing of power between patients and health professionals (42). Finally, learning about people’s lived experiences is enhanced when it is presented by the people themselves (co-education) within a sociocultural framework, as opposed to a purely medicalised perspective (41). This is crucial to facilitate learning about the importance of reasonable adjustments and adapting health assessments and management practices (33). However, it is vital to ensure that co-production is well-planned to ensure that it is not tokenistic (42) and everyone involved feels supported, especially when sharing challenging personal experiences (38).

Our team has recently adopted this co-production approach in our design, delivery, and evaluation of the GeneEQUAL Toolkit, a collection of resources that aim to improve healthcare for people with intellectual disability. We followed the six key steps of co-production recommended by the guidelines *Co-production in Action* (43), and, therefore, people with intellectual disability were included in each step of the process from the initial project idea to reflecting on the co-production process (44). People with intellectual disability reflected on how their involvement in co-production not only resulted in better resources but also was a valuable experience for them and improved their knowledge of healthcare rights (44).

## Conclusion

Despite existing legislation, there are still significant barriers and stigmatisation within the healthcare sector that limit the opportunities for people with intellectual disability to receive the highest standards of healthcare (19, 25). Co-production methodology has been used successfully, although, minimally, in the healthcare sector (42). As the GeneEQUAL team, we call for a greater emphasis on the co-production of new educational resources for health students and professionals to reduce stigmatisation and improve health outcomes for people with intellectual disability, in line with their human rights. This has the potential to facilitate a critically needed change in the model of healthcare, from one reinforcing power imbalance and trauma to an authentic partnership that is inclusive, person-centred, and respectful: indeed, a new model of co-healthcare.

## Author contributions

CM: Conceptualization, Formal analysis, Project administration, Writing – original draft. IS: Conceptualization, Methodology, Supervision, Validation, Writing – review & editing. MD: Validation, Writing – review & editing. JL: Validation, Writing – review & editing. SS: Validation, Writing – review & editing. YC-K: Validation, Writing – review & editing. MT: Validation, Writing – review & editing. JD: Validation, Writing – review & editing. SH: Validation, Writing – review & editing. SW: Validation, Writing – review & editing. JH: Validation, Writing – review & editing. TL: Validation, Writing – review & editing. BT: Supervision, Validation, Writing – review & editing. JB: Validation, Writing – review & editing. JS: Supervision, Validation, Writing – review & editing. EP: Conceptualization, Methodology, Supervision, Validation, Writing – review & editing.
