# Supplementary material for: The need for co-educators to drive a new model of inclusive, person-centred and respectful co-healthcare with people with intellectual disability
Source: Front Psychiatry. 2024 Feb 13;15:1346423. doi: 10.3389/fpsyt.2024.1346423 (PMC10896908; doi:10.3389/fpsyt.2024.1346423)
Supplement: Supplementary file 1 [file DataSheet_1.pdf]

How people with intellectual disability can teach

- **health care workers to do good health care.**

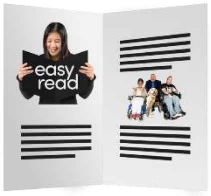

This booklet is about

- people with intellectual disability
- health care workers.

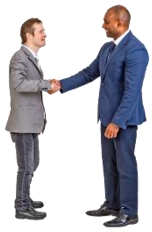

It is about how we can work together

- to make health care better.

The rules about health care for people with intellectual disability

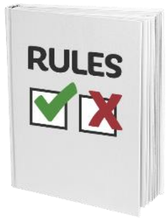

There are rules that say that people with intellectual disability

- must not be treated differently.

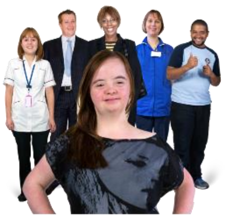

These rules say people have the right to

- good health care
- health care information they can understand.

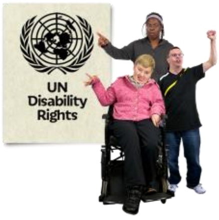

One of these rules is called

- **the United Nations Convention on the Rights of Persons with Disabilities (UNCRPD)**.

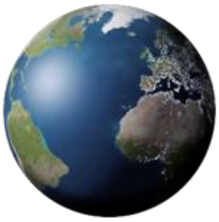

Many countries said they will follow this rule.

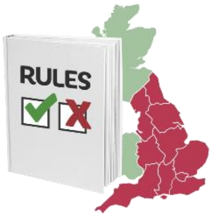

Some countries have their own rules as well.

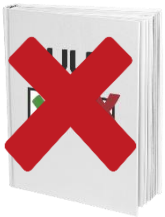

But these rules are not always followed.

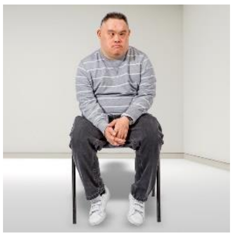

People

- can be treated badly
- can be left out
- do not have equal rights.

People can also experience abuse.

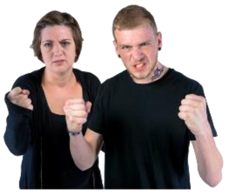

Abuse can be

- physical
    - like hitting or punching
- verbal
    - like yelling or shouting

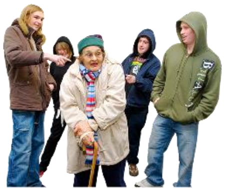

- sexual
    - like being touched when it is not wanted
- emotional
    - like saying mean things
- financial
    - like controlling money

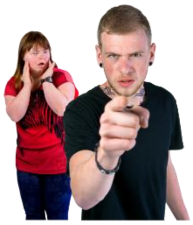

- spiritual
    - like not letting someone go to a religious place.

Health care workers can

- **treat people badly**
- **abuse people.**

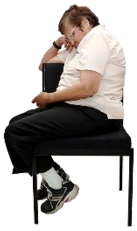

We know that people can be treated

- differently
- badly

in **health care settings**.

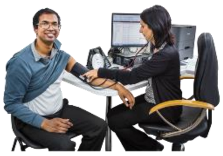

Health care settings are places

- where you get health care.

They can include

- general practitioner (GP) clinics
- hospitals
- specialist clinics
- therapy clinics.

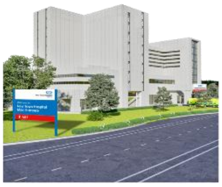

Health care workers are

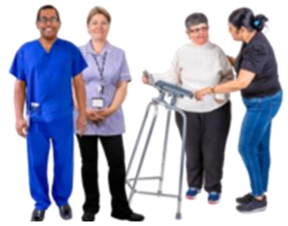

- doctors
- nurses
- other health care people.

Some health care workers think people are

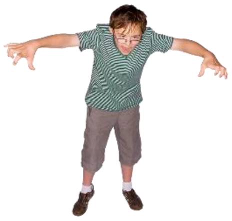

- different
- like a child
- scary

because they have intellectual disability.

Some health care workers

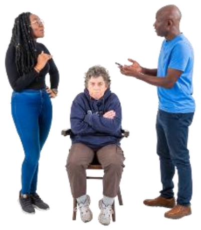

- do not believe what people tell them
- do not take new health problems seriously
- treat people differently.

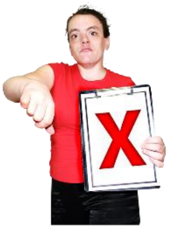

This means that people do not get health care

- that is good
- when they need it.

This can lead to

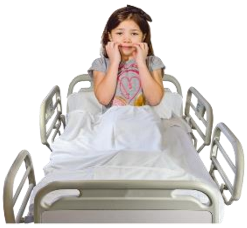

- more time spent in hospital
- poor health

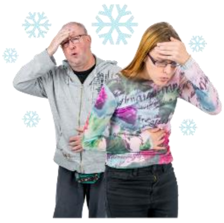

- poor mental health like
    - anxiety
    - depression
    - post-traumatic stress

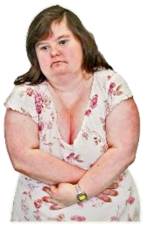

- people not having the health checks they need.

The Royal Commission is a study that

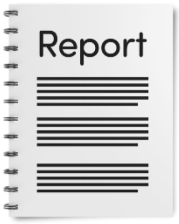

- is run by the Australian government
- listens to experiences of people with intellectual disability.

It found that people with intellectual disability can be

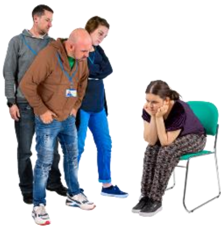

- **abused**
- **neglected**
- taken advantage of

in health care settings.

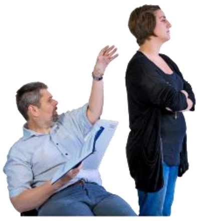

Abuse is when someone

- treats you very badly.

Neglect is when someone

- does not help you when they should.

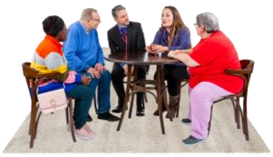

People in our GeneEQUAL research team shared

- their
- their friends

experiences in health care settings.

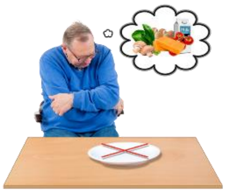

For example they shared that people were

- not allowed to make health care choices
- ignored in health care appointments
- forced to say yes to a health care choice.

Health care workers may not communicate well.

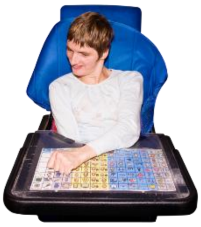

Communicate means to share information with

- words
- pictures
- signs.

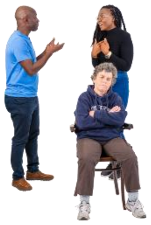

Health care workers often talk to the

- support person
- family

instead of the person with intellectual disability.

Health care workers often

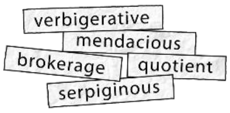

- use big words
- do not use pictures

to communicate.

This means that the person

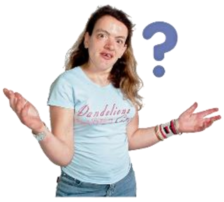

- can feel left out
- does not understand their health care information
- cannot make their own decisions.

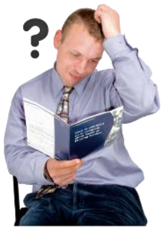

Health care information can be hard to understand.

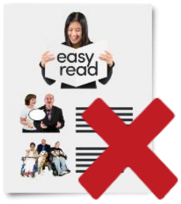

Often there is no health care information in

- Easy Read
- easy to understand pictures and videos.

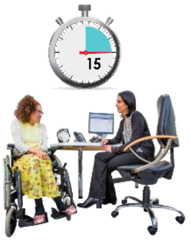

Often appointment times are

- 10 to 30 minutes
- too short for people with intellectual disability.

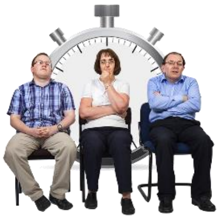

People need more time to

- ask questions
- understand health information
- make decisions.

Health care workers may not know about intellectual disability.

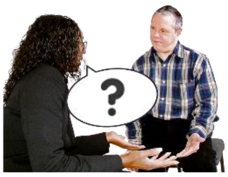

They may not know

- how to support people with intellectual disability
- about disability awareness
- the health care rules.

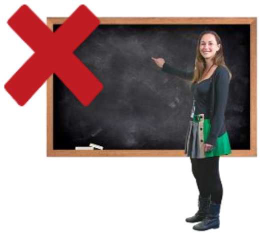

Health care workers do not learn this at

- university
- work.

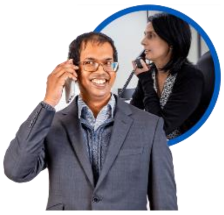

This makes it hard for health care workers

- to give good health care.

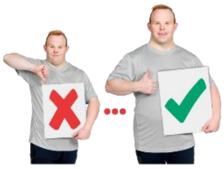

This needs to change!

We need to teach health care workers.

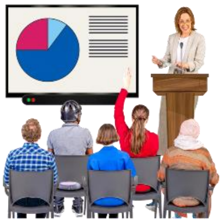

We can teach health care

- students
- workers.

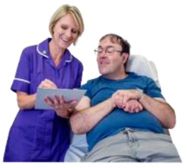

We can teach them about

- ways to support people with intellectual disability
- ways to communicate

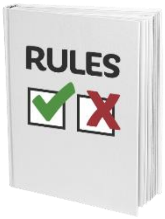

- how to make information easy to understand
- the health care rules.

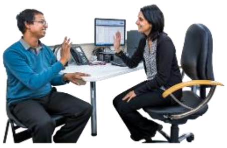

This can help health care workers

- give **good health care** to people.

In **good health care** the person

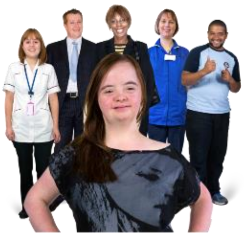

- is included as a partner
- can understand health care information
- can make their own decisions
- trusts the health care worker.

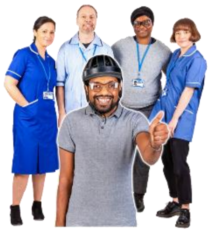

Good health care is

- inclusive
- person-centred
- respectful.

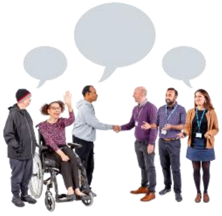

People with intellectual disability and health care workers

- can work together to teach health care workers.

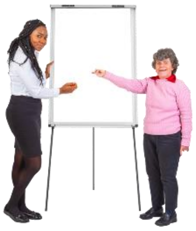

This is called co-production.

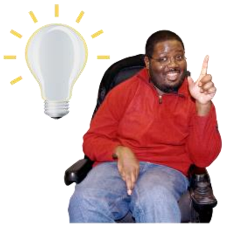

Co-production is important because people with intellectual disability

- know what works
- and what does not work

to make health care good.

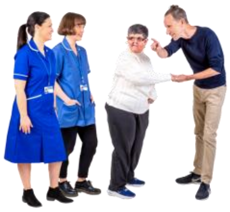

Research shows that co-production can make health care

- better
- more inclusive.

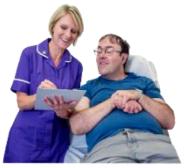

It can help health care workers

- treat people better.

Health care workers need to hear about

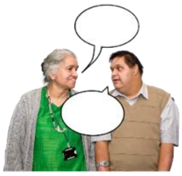

- experiences of people in health care settings.

This teaches health care workers important things like

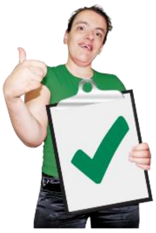

- good attitudes
- reasonable adjustments
- good communication
- easy to understand information.

What we do to help

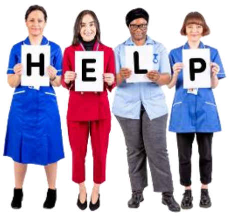

GeneEQUAL has made a Toolkit to help

- people with intellectual disability
- health care workers.

GeneEQUAL is a program to

- help people with intellectual disability

get better health care.

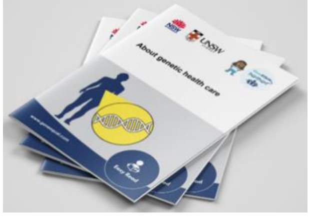

The Toolkit includes

- information for health care workers
- videos
- Easy Read booklets.

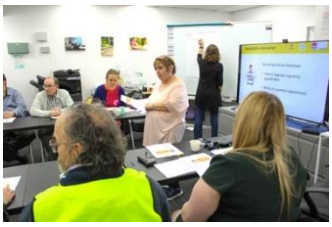

The Toolkit was made together by

- people with intellectual disability
- health care workers.

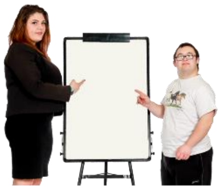

The Toolkit is an example of **co-production**.

This article is also an example of co-production.

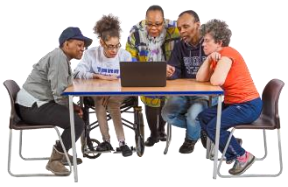

Co-production means that

- people with intellectual disability
- researchers
- health care workers

work together.

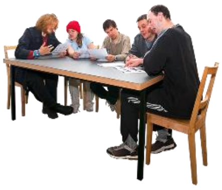

To write this article we

- read the research
- talked about the information together.

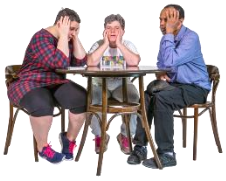

This was hard.

It brought up a lot of

- bad and difficult memories.

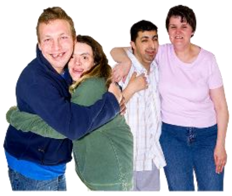

But we are a team!

We made sure that we

- looked after
- supported

each other.

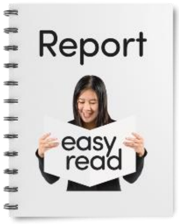

We want to share this information to

- make things better for people with intellectual disability.

The pictures in this report were from

1. the GeneEQUAL team

2. the paid photosymbols database
   https://www.photosymbols.com

PHOTO SYMBOLS
Licence Number 6032211607607
